# Detectability of Pathogenic RNA Virus Families in Different Body Sites: A Scoping Review

**DOI:** 10.3390/v18070743

**Published:** 2026-07-04

**Authors:** Christian Schaadt Ilsby, Thomas Leineweber Kristensen, Kristian Bagge, Jens Bukh, Jan Gorm Lisby, Uffe Vest Schneider

**Affiliations:** 1 Department of Virology and Microbiological Preparedness, Statens Serum Institut, 2300 Copenhagen, Denmark; thlk@ssi.dk (T.L.K.); ufvs@ssi.dk (U.V.S.); 2 Copenhagen Hepatitis C Program (CO-HEP), Department of Immunology and Microbiology, University of Copenhagen, 2200 Copenhagen, Denmark; work@kristianbagge.com (K.B.); jbukh@sund.ku.dk (J.B.); 3 Department of Clinical Microbiology, Copenhagen University Hospital—Hvidovre, 2650 Hvidovre, Denmark; jan.gorm.lisby@regionh.dk; 4 Department of Data Integration and Analysis, Statens Serum Institut, 2300 Copenhagen, Denmark; 5 Copenhagen Hepatitis C Program (CO-HEP), Department of Infectious Diseases, Copenhagen University Hospital—Hvidovre, 2650 Hvidovre, Denmark; 6 Department of Clinical Medicine, University of Copenhagen, 2200 Copenhagen, Denmark

**Keywords:** RNA viruses, viral tropism, diagnostic sampling, body sites, viral detection, scoping review

## Abstract

Nucleic acid amplification tests (NAATs) are central to modern virology diagnostics. However, evidence supporting alternative specimen types remains uneven across viral families, especially for emerging viruses. This limits diagnostic flexibility in outbreak and clinically complex settings. We conducted a scoping review of NAAT detectability across key body sites for human RNA viruses. PubMed and Embase were systematically searched for studies reporting NAAT results from urine, blood, fecal, cerebrospinal fluid, or respiratory specimens. Data were independently screened and synthesized to summarize specimen-specific detectability for each virus. From 8676 screened records, 321 studies were included, covering 39 viruses across 25 RNA virus families. Detectability across specimen types varied substantially between viruses. Consistent detection across multiple specimens was observed for few viruses, including SARS-CoV-2, Zika virus, and HIV, whereas many emerging viruses were evaluated in a single body compartment with limited comparative data. NAAT performance across specimen types is highly virus-specific and unevenly studied, with reliance on blood or respiratory specimens, potentially overlooking viable, less invasive alternatives. Evidence gaps are particularly pronounced for urine and cerebrospinal fluid, and heterogeneous reporting limits cross-study comparability. Standardized, cross-specimen and longitudinal studies are needed to improve diagnostic strategies, outbreak preparedness, and future assay development.

## 1. Introduction

Nucleic acid amplification tests (NAATs), particularly polymerase chain reaction (PCR), have transformed the field of virology by providing a highly sensitive and specific method for confirming the presence of viruses. PCR has become a cornerstone in clinical infectious disease diagnostics, allowing for the rapid and accurate identification of viral pathogens from a variety of biological specimens. This technology plays a pivotal role in early diagnosis, treatment monitoring, and epidemiological surveillance, significantly improving patient outcomes and public health responses [1].

Across virology, alternative specimen types for virus diagnostics are actively studied, but the depth and quality of this work are uneven—particularly for emerging human viruses [2,3]. In practice, most NAAT-based research continues to rely on well-established specimen types known to perform consistently, leaving gaps in knowledge that likely vary substantially between viral families [4].

Viral RNA may be detectable in specimens beyond the primary site of infection owing to systemic dissemination, viral shedding into bodily fluids, and persistence of viral nucleic acids. Consequently, detectability may vary substantially across specimen types and stages of infection [5,6,7]. Alternative specimen studies may provide valuable diagnostic information in clinically complex cases. This can be true when delayed diagnostic clarity means that only non-standard specimen types are available during the relevant disease window. Additionally, expanding sampling coverage within known viral families is important for future screening programs, since virus discoveries are expected to predominantly fall within these rather than representing novel families [8,9,10].

Across viruses and specimen types substantial heterogeneity in study design, reporting practices, detectability, and data presentation is present. Due to these differences as well as the quantity of studies identified, a scoping review was considered the most appropriate approach to meaningfully characterize the existing evidence and identify gaps in knowledge. Accordingly, this scoping review aims to provide a comprehensive overview of the detectability and diagnostic utility of NAATs across all RNA virus families affecting human patients. Additionally, we will focus on identifying the evidence supporting the use of specimens from different body sites for viral detection. By synthesizing current knowledge on NAAT performance across specimen types and highlighting areas of limited or uneven evidence, this review seeks to inform future research priorities and may potentially support improved diagnostic strategies in outbreak and clinically complex settings.

## 2. Materials and Methods

This study was conducted in accordance with the Preferred Reporting Items for Systematic reviews and Meta-Analyses extension for Scoping Reviews (PRISMA-ScR) (Table S1) [11,12]. The review was prospectively registered on PROSPERO (CRD42024608133).

This scoping review was conducted according to the Joanna Briggs Institute (JBI) methodology for scoping reviews and structured using the Population–Concept–Context (PCC) framework. The population comprised human cases with confirmed RNA virus infection, the concept was detectability by NAAT and the context was clinical diagnostic specimens. The review question was: “What evidence exists regarding the detectability of human pathogenic RNA viruses by NAAT across different clinical specimen types, and where are the current gaps in knowledge?”

### 2.1. Viruses and Specimen Types

All RNA viruses found in humans and related to human disease were examined in the study with at least one representative virus for each family [13,14]. As such, 25 RNA virus families with 39 relevant viruses were included. Families more commonly associated with hospital diagnostics have several viruses included in the scoping review. Additionally, due to the impending reorganization of *Flaviviridae*, we found it prudent to accommodate the changes in advance [15].

As we intend to cover all major body sites, we included clinical urine, blood, fecal, central nervous system (CNS) specimens, or respiratory specimens as well as related specimen types. Related specimen types for each body site are:

- Blood specimens: whole blood, serum, plasma, and PBMC;
- Respiratory specimens: saliva, bronchoalveolar lavage (BAL), oropharyngeal swabs, nasopharyngeal swabs, nasal swabs, and oral swabs, sputum;
- Fecal specimens: stool and rectal swabs;
- Urine specimens: urine, urinary sediment, and urine supernatant;
- CNS specimens: cerebrospinal fluid (CSF).

### 2.2. Search Strategy and Selection Criteria

Through systematic searches of PubMed and Embase using Medical Subject Heading (MeSH), Emtree, and title/abstract terms, we retrieved research articles according to the predefined PCC framework and eligibility criteria for each of the 39 included viruses. Full search strategies are provided in Table S2. We included randomized controlled trials, cohort studies, case–control studies, diagnostic accuracy studies, cross-sectional studies, case series and case reports that fulfilled the eligibility criteria. Subsequently, the reference lists of included studies and relevant excluded reviews were also screened for additional studies.

Given the broad scope of the review, encompassing 39 viruses across 25 RNA virus families, measures were prespecified to maintain feasibility while prioritizing studies providing comparative information relevant to the review objective. When a specific virus–specimen search combination yielded more than 500 records, the search strategy was refined to preferentially identify studies evaluating that specimen together with one or more additional specimen types. For example, if a virus–blood specimen search exceeded the threshold, blood-related search terms were combined with at least one additional specimen category, whereas searches involving less frequently studied specimens remained unchanged. This approach reduced retrieval of studies examining only established reference specimens while retaining studies capable of informing cross-specimen detectability. The threshold of 500 records was selected a priori as a pragmatic limit to maintain feasibility. Full search strategies are provided in Table S2.

During full-text screening, studies were prioritized according to a prespecified hierarchy favoring (i) evaluation of multiple specimen types within the same patients, (ii) larger patient cohorts, and (iii) longitudinal sampling. When multiple studies provided overlapping information for a given virus–specimen combination, studies with greater comparative value were retained while less informative studies were excluded according to the hierarchy (Appendix A, Table A1). This approach was intended to maximize the breadth of specimen-level evidence while maintaining a feasible review process.

### 2.3. Eligibility Criteria

Inclusion:

- Human cases with confirmed infection by a virus of interest.
- Viral nucleic acid testing (NAAT) performed.
- Clinical specimens of urine, blood, feces, cerebrospinal fluid, or respiratory material collected for diagnostic purposes.

Exclusion:

- Duplicate patient data published elsewhere.
- Studies limited to post-mortem, asymptomatic screening, neonates, immunocompromised patients, pregnant persons, or antivirally treated cases prior to NAAT testing.
- Studies primarily comparing a novel non-NAAT assay against a standard NAAT.
- Reviews and conference abstracts.

While immunocompromised patients are excluded, human immunodeficiency virus (HIV) was included in study screening. As such, individuals with HIV were excluded if they were considered severely immunodeficient, defined as having a CD4 count below 200 cells/µL or a CDC clinical category of C or higher.

### 2.4. Data Extraction

Two authors (CI and TK) independently charted data by using two non-included DNA-viruses mpox virus and adenovirus to determine software choice and extraction variables [12]. Afterwards, three authors (CI, TK and KB) independently screened and retrieved articles according to the eligibility criteria, reviewed full-text articles for final inclusion in the review, and performed the subsequent data extraction and critical appraisal. Each data point of screening selection and data extraction had a minimum of two people assessing. Any disagreements regarding study inclusion or data were resolved through discussion between the three authors—CI had final say. Extracted data contains as a minimum: distribution of age, distribution of sex, number of cases, number of detected cases (C_d_) compared to total cases (C_t_), type of NAAT used, examined specimen types (and related specimens), and presence of data on detectability over time. If able, the authors extracted data on time to viral clearance or last day reported test-positive. If virus findings were not reported per individual and/or specimen types were not described, the study was excluded due to “wrong study design”. If specific data items were missing or unclear, it was logged as “not extractable”. If the method of diagnosis for the intended virus was unclear or different from NAAT the article was excluded due to “wrong detection method”. All study screening, data extraction and appraisal was performed using the Covidence platform (Covidence systematic review software, Veritas Health Innovation, Melbourne, Australia. Available at www.covidence.org).

### 2.5. Synthesis of Evidence

Descriptive synthesis of evidence was performed for the studies included. For each study, each examined specimen had its C_d_/C_t_ proportion logged for each virus of interest. The C_d_/C_t_ proportions were subsequently presented as percentage value ranges for each virus and each specimen type. Subsequently for each virus and specimen type an overall detectability estimate (D) was given by the authors for each based on the relationship between C_d_ and C_t_ of the individual studies and their hierarchy categories (Table 1). The certainty of each D estimate was qualitatively assessed according to the volume and strength of available evidence, including (i) number of studies, (ii) hierarchical study quality, and (iii) total number of examined cases. Virus–specimen combinations supported by sparse evidence (e.g., few studies, low hierarchical rating, and/or small cumulative sample size) were labeled as uncertain and are indicated by hatched shading in Table 1. For viruses where NAAT in a specific specimen type constitutes the diagnostic reference standard, detection in that specimen was inherently near 100%, as case confirmation was contingent upon positivity in that specimen. The studies included were categorized according to the following study characteristic: (1) virus examined, (2) specimen types examined, (3) longitudinal data, and (4) hierarchical category (Table S3).

## 3. Results

**Figure 1:**
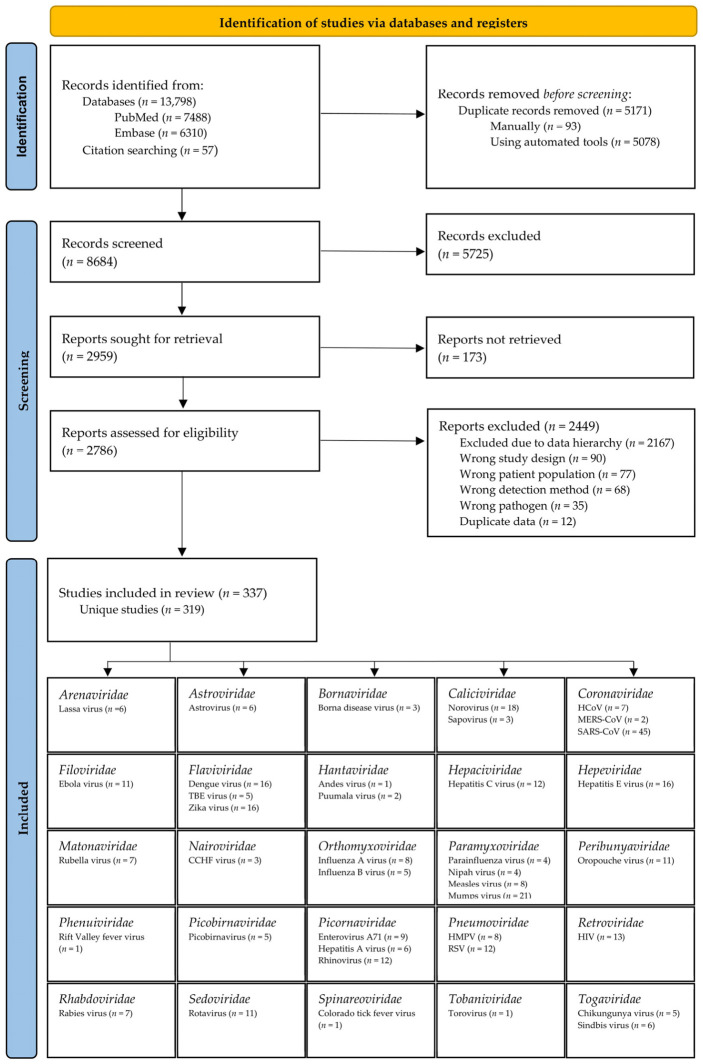
Inclusion flowchart. Inclusion flowchart showing the 319 included studies in the review. Eight studies contained data for multiple different viruses and had data extracted for each relevant virus.

In the scoping review 8676 titles and abstracts were screened with 2959 articles sought for retrieval. Based on our eligibility criteria, 319 unique studies were included throughout all the 25 virus families (Figure 1).

Of the 319 studies included, 8 contained data relevant for several viruses [16,17,18,19,20,21,22,23]. A total of 161 studies contained data on consecutive patient sampling for the included virus. A sum of 102 studies were graded A in hierarchical screening for two or more specimen types (Table S3). A total of 27 studies contained data on at least four different specimen types [22,24,25,26,27,28,29,30,31,32,33,34,35,36,37,38,39,40,41,42,43,44,45,46,47,48,49], with 3 studies containing data on all five [50,51,52].

**Figure 2:**
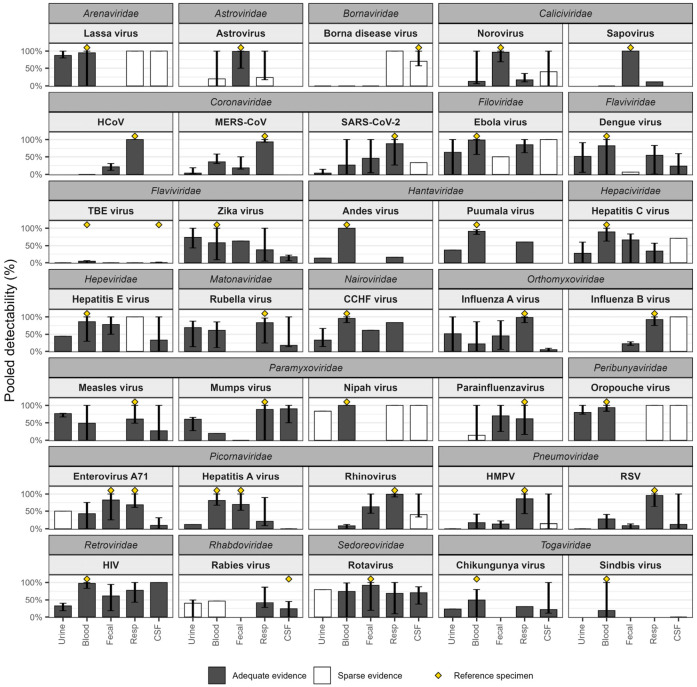
Pooled detectability estimates across specimen types. Bars represent pooled detectability calculated from cumulative detected cases (C_d_) divided by cumulative tested cases (C_t_). Whiskers indicate the range of study-level detectability estimates. Open bars indicate virus–specimen combinations supported by sparse evidence. Yellow diamond indicates specimen commonly used as reference specimen. Abbreviations: Resp; respiratory specimens.

For each virus, the specimen type constituting the diagnostic reference standard was identified where applicable and is indicated in Table 1 and Figure 2. Detectability estimates for alternative specimens were interpreted relative to these reference-standard specimens.

Within *Arenaviridae* Lassa virus was analyzed and had all specimen types except fecal specimens reported. Detection outside the blood reference standard was reported mainly in isolated case reports with urine appearing an option despite low total tested cases (Range: 80–100%).

*Adenoviridae* was almost exclusively examined in the reference standard fecal specimens. Blood or respiratory material studies trended poorly in detection for human astrovirus (C_d_/C_t_: 1/5, 6/25) but data is sparse and insufficient.

*Bornaviridae* was only described in small series or individual cases, with CSF as the primary diagnostic material and only a single case report examining other specimens than CSF or blood [50].

For *Caliciviridae*, fecal specimens are the reference standard. Norovirus was well characterized in blood and respiratory specimens but the performance was poor (C_d_/C_t_: 50/388, 66/384). Sapovirus remained limited to fecal surveillance with low detection in other specimens across two studies [16,19]. Urine specimens were never assessed for this family.

Among *Coronaviridae*, respiratory specimens were the reference standard. Human coronaviruses OC43, NL63, HKU-1 and 229E (HCoV) were sparsely studied beyond respiratory specimens but, from the sparse literature on fecal detection, were found low and viremia never detected (C_d_/C_t_: 0/58) [17,53,54]. Middle east respiratory syndrome-coronavirus (MERS-CoV) was evaluated in two comprehensive case series with similar findings; however, there were no reports on CNS testing even in severe cases [24,25]. Due to the international focus, severe acute respiratory syndrome-coronavirus-2 (SARS-CoV-2) had been comprehensively assessed across all specimen types, even occasionally for CSF in severe disease with mixed findings (C_d_/C_t_: 4/12) (Table 1).

**Table 1:** Ranges of detectability across five specimen types.

Family	Virus	Urine				Blood				Respiratory				Cerebrospinal				Fecal			
		D	Range	C_d_/C_t_	References	D	Range	C_d_/C_t_	References	D	Range	C_d_/C_t_	References	D	Range	C_d_/C_t_	References	D	Range	C_d_/C_t_	References
*Arenaviridae*	Lassa virus		80–100%	7/8	[55,56,57]	*	0–100%	39/41	[55,56,57,58,59,60]		100%	2/2	[57]		100%	2/2	[58,60]		-	-	-
*Astroviridae*	Human astrovirus		-	-	-		0–100%	1/5	[19,61]		17–100%	6/25	[16,61]		-	-	-	*	50–100%	101/102	[16,19,61,62,63,64]
*Bornaviridae*	Borna disease virus		0%	0/1	[50]		0%	0/8	[50,65]		100%	1/1	[50]	*	57–100%	7/10	[50,65,66]		0%	0/1	[50]
*Caliciviridae*	Norovirus		-	-	-		6–100%	50/388	[67,68,69,70,71,72]		12–35%	66/384	[16,73,74]		0–100%	2/5	[67,68,72,75]	*	69–100%	794/819	[16,67,68,69,70,71,72,73,74,75,76,77,78,79,80,81,82,83]
*Caliciviridae*	Sapovirus		-	-	-		0%	0/10	[19]		12%	5/43	[16]		-	-	-	*	100%	148/148	[16,19,84]
*Corona-* *viridae*	HCoV		-	-	-		0%	0/58	[17,53,54]	*	100%	775/775	[17,18,53,54,85,86,87]		-	-	-		11–31%	19/89	[18,53,54]
*Corona-* *viridae*	MERS-CoV		2–19%	7/185	[24,25]		31–58%	47/132	[24,25]	*	93–100%	218/232	[24,25]		-	-	-		15–50%	17/92	[24,25]
*Corona-* *viridae*	SARS-CoV-2		0–14%	18/479	[26,27,28,29,30,31,32,33,88,89,90,91,92,93,94]		0–100%	141/536	[26,29,30,32,33,93,94,95,96,97,98,99,100,101,102,103]	*	26–100%	2613/2949	[26,27,28,29,30,31,32,33,88,89,90,91,92,93,94,95,96,97,98,99,101,102,103,104,105,106,107,108,109,110,111,112,113,114,115,116,117,118,119,120,121]		33%	4/12	[103]		4–100%	1040/2265	[26,27,28,29,30,31,32,33,88,89,90,91,94,95,96,97,98,99,100,101,102,103,104,105,106,107,108,109,110,111,112,113,114,115,116,117,118,119,120,121,122,123,124,125]
*Filoviridae*	Ebola virus		0–100%	14/22	[34,35,126,127]	*	57–100%	810/820	[34,35,126,127,128,129,130,131,132,133,134]	*	62–100%	53/62	[34,35,127,132]		100%	2/2	[34,129]		50%	2/4	[35]
*Flaviviridae*	Dengue virus		6–92%	398/777	[23,135,136,137,138,139,140,141,142,143,144,145]	*	0–100%	632–766	[23,135,136,138,139,140,141,142,143,144,145,146,147,148,149]		1–83%	316–574	[135,136,139,141,142,143,144,145]		0–60%	8/34	[146,147,148,149]		6%	2/32	[137]
*Flaviviridae*	TBE virus		0%	0/11	[150]	*	0–6%	13/281	[37,150,151,152,153]		0%	0/15	[150,153]	*	0–2%	1/220	[151,152,153]		0%	0/2	[37]
*Flaviviridae*	Zika virus		43–100%	676/912	[23,38,154,155,156,157,158,159,160,161,162,163,164,165]	*	9–100%	643/1103	[20,23,38,154,155,156,157,158,159,160,161,162,163,164,165,166]		5–100%	279/734	[38,154,155,157,160,161,162,164]		6–22%	21/122	[20,166]		63%	165/260	[38]
*Hantaviridae*	Andes virus		13%	8/60	[167]	*	100%	131/131	[167]		16%	21/131	[167]		-	-	-		-	-	-
*Hantaviridae*	Puumala virus		37%	25/67	[168]	*	82–96%	91/100	[168,169]		61%	20/33	[169]		-	-	-		-	-	-
*Hepaci-* *viridae*	Hepatitis C virus		0–60%	61/222	[36,170,171,172,173,174]	*	63–100%	377/422	[36,170,171,172,173,174,175,176,177,178,179,180]		0–57%	62/180	[36,170,171,172,173,175,176]		71%	5/7	[177]		0–83%	82/123	[36,178,179,180]
*Hepeviridae*	Hepatitis E virus		44%	4/9	[181]	*	30–100%	128/149	[181,182,183,184,185,186,187,188,189,190,191,192,193,194,195,196]		100%	8/8	[192]		0–100%	6/18	[182,183,185,188,189,190,191,194,195,196]		50–100%	84/108	[184,185,186,187,189,193,194]
*Matona-* *viridae*	Rubella virus		14–87%	349/506	[197,198,199,200]		11–85%	362/594	[197,199,200,201,202,203]		25–96%	533/642	[197,199,200,201,202,203]		14–100%	4/22	[21,203]		-	-	-
*Nairoviridae*	CCHF virus		15–67%	22/66	[39,204,205]	*	83–100%	63/66	[39,204,205]		83%	15/18	[39]		-	-	-		61%	11/18	[39]
*Orthomyxoviridae*	Influenza A virus		0–100%	30/58	[40,41,42,51]		0–86%	25/112	[40,41,42,51,206,207]	*	83–100%	160/163	[40,41,42,51,206,207,208,209]		0–9%	1/20	[51,206,209]		6–89%	40/88	[40,41,42,51,208]
*Orthomyxoviridae*	Influenza B virus		-	-	-		-	-	-	*	75–100%	177/192	[18,210,211]		100%	2/2	[212,213]		18–28%	30/132	[18,210]
*Paramyxoviridae*	Measles virus	*	67–77%	506/661	[214,215,216]		0–100%	81/166	[202,214,215,217,218]	*	49–100%	149/245	[202,214,215,216,217,218,219]		0–100%	17/62	[202,216,217,218,219,220]		-	-	-
*Paramyxoviridae*	Mumps virus		28–65%	136/226	[221,222,223,224,225,226]		20%	23/116	[222]	*	0–100%	363/412	[221,222,223,224,225,226,227,228,229]		50–100%	173/192	[21,221,223,227,228,229,230,231,232,233,234,235,236,237,238,239,240]		0%	0/1	[229]
*Paramyxoviridae*	Nipah virus		83%	5/6	[241]		100%	19/19	[242,243]		100%	7/7	[242,244]		100%	1/1	[242]		-	-	-
*Paramyxoviridae*	Human parainfluenza virus		-	-	-		0–100%	1/7	[17,245]	*	17–100%	8/13	[17,18,245]		-	-	-		25–100%	7/10	[18,246]
*Peribunyaviridae*	Oropouche virus		75–100%	4/5	[247,248]	*	83–100%	291/311	[247,248,249,250,251,252,253,254,255]		100%	2/2	[248]		100%	6/6	[256,257]		-	-	-
*Phenuiviridae*	Rift Valley fever virus		-	-	-	*	79%	49/62	[258]		-	-	-		-	-	-		-	-	-
*Picobirnaviridae*	Picobirnavirus		-	-	-		-	-	-		-	-	-		-	-	-		100%	51/51	[259,260,261,262,263]
*Picornaviridae*	Enterovirus A71		50%	1/2	[52]		0–76%	165/387	[43,44,45,46,52,264]	*	61–100%	207/301	[43,44,45,46,52,265,266,267]		0–31%	28/290	[43,44,45,46,52,264,266,267]	*	25–100%	435/525	[43,44,45,46,52,264,265,266,267]
*Picornaviridae*	Hepatitis A virus		12%	8/65	[268]	*	68–100%	105/129	[47,269,270,271,272]		9–90%	14/67	[268,271]		0%	0/120	[47]	*	52–100%	83/119	[47,269,270,272]
*Picornaviridae*	Rhinovirus		-	-	-		0–12%	114/1369	[17,273,274,275,276]	*	91–100%	1949/1961	[17,18,273,274,275,276,277,278,279,280,281]		33–100%	4/10	[280,282]		44–100%	186/295	[18,280,281]
*Pneumoviridae*	HMPV		0%	0/6	[22]		0–41%	7/41	[17,22,283]	*	43–100%	75/87	[17,18,22,283,284,285,286,287]		0–100%	1/7	[284,286,287]		0–22%	2/15	[18,22]
*Pneumoviridae*	RSV		0%	0/33	[22]		0–40%	41/148	[17,22,288,289]	*	63–100%	381/398	[17,18,22,288,289,290,291,292,293,294,295]		0–100%	2/16	[290,291,296]		0–14%	5/57	[18,22]
*Retroviridae*	HIV		18–40%	10/31	[297,298]	*	84–100%	555/569	[297,298,299,300,301,302,303,304,305,306,307,308,309]		43–100%	113/145	[301,302,304,305,306,307]		100%	77/77	[305,308]		18–95%	182/295	[297,299,300,303,309]
*Rhabdoviridae*	Rabies virus		0–50%	2/5	[48,310]		47%	7/15	[48]		28–87%	71/169	[48,310,311,312,313,314,315]	*	0–45%	26/109	[48,310,311,312,313,314,315]		-	-	-
*Sedoreoviridae*	Rotavirus		80%	4/5	[316]		0–99%	243/326	[49,317,318,319,320,321,322,323]		10–100%	396/574	[16,49,324]		38–88%	17/24	[49,317,320]	*	19–100%	817/890	[16,49,316,318,319,320,321,322,323,324]
*Spinareoviridae*	Colorado tick fever virus		-	-	-	*	100%	11/11	[325]		-	-	-		-	-	-		-	-	-
*Tobaniviridae*	Torovirus		-	-	-		-	-	-		-	-	-		-	-	-		100%	18/18	[326]
*Togaviridae*	Chikungunya virus		23%	35/152	[327]	*	0–80%	138/277	[20,146,327,328,329]		30%	46/152	[327]		11–100%	15/70	[20,146,327,328,329]		-	-	-
*Togaviridae*	Sindbis virus		-	-	-	*	0–100%	31/166	[330,331,332,333,334]		-	-	-		0%	0/1	[335]		-	-	-

D: overall detectability estimate. Dark green: high detectability (>75%); light green: medium detectability (25–75%); yellow: low detectability (<25%); red: very low detectability/undetectable (<5%); hatched shading: uncertain estimate; grey: non-existent literature; Range: range from lowest included to highest of included studies’ average detectability of confirmed cases out of total cases reported; Ref: references of included studies; C_d_: detected patient cases; C_t_: total confirmed cases. * Reference specimen.

Ebola virus detection used blood as the reference standard, but detection was also frequent across other specimen types, though systematic assessment was limited by general outbreak conditions (Table 1) [4].

In the new *Hepaciviridae*, for hepatitis C virus, blood was the reference standard and the other four specimen types appeared as possible alternative options—although data was conflicting [36,172,173,179].

*Flaviviridae* used blood as the reference standard specimen for the included viruses. Tick-borne encephalitis (TBE) virus was found with sparse data on NAAT detectability, with testing rarely performed outside blood and CSF and positive findings in non-blood specimens reported infrequently (C_d_/C_t_: 13/281, 1/220) [37,151,153]. In contrast, Zika virus and dengue virus were comprehensively studied in large cohorts and demonstrated consistently broad detectability across specimen types, indicating that urine and saliva as alternatives to blood are clearly possible (Figure 2) [38,155,162]. For both viruses, blood and occasionally saliva showed high detectability early in the course of infection, whereas, for dengue virus, urine consistently exhibited lower detectability initially but remained detectable for a longer duration than other specimens [38,135,139,143,144,145,162,164]. However, compared with Zika virus, dengue virus was only assessed to a limited extent in fecal specimens across the available literature [137].

For *Hantaviridae*, Andes virus and Puumala virus were included and blood was the reference standard for both. Urine and respiratory specimens appear supplementary but inferior options (Table 1) with fecal or CSF specimens were never described [167,168,169].

*Hepeviridae’s* hepatitis E virus displayed high detectability in both blood and fecal specimens, which were interchangeably used as the reference standard. Detection in other materials was infrequent and typically single-case.

*Matonaviridae’s* rubella virus was most consistently identified in respiratory specimens (Range: 25–95%), with urine and blood appearing very useable despite large variation in reported findings (Range: 14–87%, 11–85%).

For *Nairoviridae’s* Crimean-Congo hemorrhagic fever (CCHF) virus, blood was the reference standard, yet RNA was varyingly detected across urine (C_d_/C_t_: 15/18), respiratory (C_d_/C_t_: 15/18), and fecal samples (C_d_/C_t_: 11/18), suggesting broader shedding potential in the few published studies.

Among *Orthomyxoviridae*, influenza A virus and influenza B virus used respiratory specimens as the reference standard. Influenza A virus had substantial variability in urine (Range: 0–100%), blood (Range: 0–86%), and fecal (Range: 6–89%) findings. Conversely, influenza B virus was across few studies only examined in respiratory and fecal specimens, with the latter having poor detectability (Range: 18–28%) [18,210].

*Paramyxoviridae* similarly relied primarily on respiratory specimens for detection. Measles virus and mumps virus inherently suffered in inclusion due to the prevalence of the MMR vaccine. While mumps virus had few outbreaks large enough for proper examination, measles virus had sufficient spread in populations to allow for thorough mapping with multiple options for NAAT (Figure 2). Urine (Range: 67–77%) appears at minimum on par with blood and respiratory specimen for measles virus detection. Nipah viruses were sparsely reported, likely due to the same factors that affect *Filoviruses* and similar viruses. However, from the limited data all examined body sites appear options for testing [241,242,243,244]. Human parainfluenza viruses were too sparsely reported but have been attempted in small-scale fecal samples and blood [17,18].

For *Peribunyaviridae* and Oropouche virus, available evidence is largely restricted to the blood reference standard, with a few case reports examining RNA presence in urine, CSF, and respiratory specimens.

*Phenuiviridae* studies were sparse and confined solely to blood detection.

*Picobirnaviridae* research was found to focus on fecal detection in diarrheal cases with clear association to disease not yet established (Table 1).

*Picornaviridae* are generally well characterized as a group. For Enterovirus A71 a consistent reference was not used and RNA were detected in all specimen types, with the highest rates in fecal (C_d_/C_t_: 435–525) and respiratory specimens (C_d_/C_t_: 207–301). Rhinovirus used respiratory specimens as the reference standard with moderate fecal specimen detectability (C_d_/C_t_: 186/295) and blood appearing a poor option (C_d_/C_t_: 114/1369). Hepatitis A virus used blood and fecal specimens interchangeably as reference standards with only respiratory samples appearing an alternative option (C_d_/C_t_: 14/67).

For *Pneumoviridae* respiratory specimens were used as the reference standard and other specimens were rarely examined—no notable alternatives appear from these studies (Table 1) [22].

HIV used blood as the reference standard and was detectable in all examined specimen types, consistent with its systemic nature. HIV was always detected in CSF in the few studies we included that examined the specimen (C_d_/C_t_: 77/77) [305,308]. Urine and, to a lesser extent, fecal specimens were the least studied and poorest options (Table 1 and Figure 2).

Within *Rhabdoviridae* rabies virus detection relied primarily on saliva and CSF, with post-mortem brain tissue often acting as reference standard—other body sites were sparse and/or non-existent in the literature (Table 1).

*Sedoreoviridae* and rotavirus were extensively characterized with fecal specimen as reference. Detection was described in all body sites, with blood and respiratory specimens having variable but moderate values (C_d_/C_t_: 243/326, 396/574). Colorado tick fever virus was reported on sparsely and only in blood [325].

*Tobaniviridae* was poorly described, with a single PCR study detecting RNA in diarrheal stool [326].

Finally, within *Togaviridae,* chikungunya virus used blood PCR as the reference standard and was examined across serum, urine, and saliva in a large cohort study [327]. Sindbis virus, though historically present in Northern Europe and Russia, was only detected in blood using NAAT and remains underreported. Serological confirmation or skin biopsy PCR were interchangeably reference standards and allowed for more clear estimates of blood NAAT useability values in the absence of other body sites (C_d_/C_t_: 31/166).

## 4. Discussion

As a scoping review, the objective was to map the breadth and distribution of evidence rather than generate pooled estimates of diagnostic performance or comparative accuracy between specimen types.

Previous reviews on viral detectability have attempted similar mappings of the literature across various groups of viruses; however, the reviews are very often limited to a small select handful—often viruses with similar symptomatology. During the COVID-19 pandemic, Cevik et al. 2021 reported an in-depth analysis of the literature across the three most recently outbreak-related coronaviruses [6]. This scale allowed for more detailed analysis on estimates such as mean shedding duration across specimen types. Lowry et al. 2023 attempted a similar mapping of the literature on common respiratory viruses and their shedding profiles in human patients—although on a smaller scale [336]. In that study the focus was more to improve the patient estimates from wastewater virus concentrations rather than creating an overview of viral families [336].

Across the examined RNA virus families, patterns of detectability varied considerably between specimen type, although certain consistent patterns emerged. Sixteen viruses had studies assessing NAAT detectability across all five specimen categories included in this review (Table 1 and Table S3). In contrast, several viruses were characterized by limited evidence, reflected by a low number of studies, small cumulative sample sizes, and/or restricted diversity of specimen types examined. These included Lassa virus, human astrovirus, Borna disease virus, sapovirus, HCoV, influenza B virus, Nipah virus, human parainfluenza virus, picobirnavirus, Sindbis virus, torovirus, Colorado tick fever virus, Rift Valley fever virus and enterovirus A71 (Table 1 and Figure 2).

The reasons for these evidence gaps likely differ between viruses. For outbreak-associated viruses such as Lassa virus, Nipah virus, and Ebola virus limited data may reflect sporadic outbreaks, biosafety requirements, high mortality, and concentration of cases in resource-constrained settings [4,337,338,339]. For Ebola virus specifically, much of the detailed literature on viral persistence and shedding concerns convalescent patients and therefore fell outside the scope of the present review [340,341]. In contrast, TBE virus illustrates how disease biology may limit NAAT-based investigations, as its brief viremic phase is often followed by neurological manifestations weeks after viral clearance, reducing the utility of molecular diagnostics and likely contributing to the sparse detectability data identified here [342,343]. Human parainfluenza virus and similar respiratory viruses were primarily examined in respiratory panel surveillance studies, where relatively mild disease, underdiagnosis, competition for research funding, and the absence of licensed antiviral therapies may have limited broader investigation [344,345,346]. Finally, for Borna disease virus, previously contested reports linking viral detection to psychiatric illness in cross-sectional screenings of otherwise healthy individuals were excluded based on the review eligibility criteria [347,348].

Not all evidence gaps were confined to rare or neglected viruses. Even among several well-studied viruses commonly investigated in resource-rich settings, data on NAAT detectability in urine were sparse or entirely absent despite the ease and non-invasive nature of specimen collection. Examples include norovirus, hepatitis E virus, HMPV, and RSV (Table 1). This suggests that specimen selection in virological research may often be guided by established diagnostic practice rather than systematic evaluation of alternative specimen types.

For a certain group of viruses with no natural convalescent period longitudinal sampling served other purposes than measuring speed of clearance—examples include hepatitis C virus, HIV, and rabies virus. For rabies virus, and the other viruses historically, the continued worsening, lack of treatment and often resource-poor setting makes duration of shedding irrelevant and relegates body site clearance monitoring to a solely academic exercise [349]. Another inherent limitation in the HIV inclusion is that older studies with cases untreated over long periods or debut cases never having received treatment met exclusion criteria for immunosuppression—this limited study inclusion to patients from resource-poor settings without access to antiretroviral therapy (ART), studies from the pre-ART era or acute HIV patients.

Figure 2 illustrates substantial variation in detectability patterns across viruses and specimen types. For several viruses, including rotavirus, Nipah virus, Ebola virus, Lassa virus and Zika virus, detectability was generally high across multiple specimen types, whereas respiratory viruses such as influenza A virus, influenza B virus, RSV, HMPV, SARS-CoV-2 and HCoVs showed greater differences between specimen types. Wide ranges for some virus–specimen combinations reflect considerable between-study heterogeneity.

The five specimen types included in this review were selected based on their clinical relevance and scalability. Specimens such as urine and respiratory material are easily collected and readily implemented in large-scale diagnostic or screening settings. Urine already makes up the largest part of culture-based tests in the clinical microbiology laboratories. Additionally, it can be obtained non-invasively without specialized personnel or facilities and may serve as a route of viral elimination even for pathogens without a primary urogenital focus [2]. Respiratory specimens, such as nasopharyngeal or oropharyngeal swabs, sputum, and saliva are similarly accessible and have been of particular importance in epidemic and pandemic surveillance due to the tropism of historic outbreaks [350].

Blood sampling, although more invasive, is routinely performed for patients with suspected infections in most hospitals, making it a practical diagnostic specimen [2]. Fecal material, while slightly less convenient to collect, remains clinically relevant for enteric and systemically shedding viruses [351]. In contrast, CSF sampling is invasive and typically reserved for severe or neurologically focused disease. Nevertheless, it remains clinically important for neurotropic infections and was therefore included despite its more limited availability. Other specimen types, such as skin swabs, breast milk, or semen, were not included due to their limited representation in the literature and, for some, the logistical challenges associated with large-scale collection.

Our search strategy permits us to map the detectability of viruses with very different levels of evidence by being able to limit certain single-specimen studies when the amount is very large in order to then collect studies with multiple specimen types and subsequently categorizing the extent to which the patients were examined across multiple specimen types. These approaches ensure that our review remains comprehensive by both having smaller reports available for sparsely described viruses at the same time as not including overwhelming amounts for very well published viruses.

A strength of this paper is the mapping of large studies describing NAAT detection in consecutive sampling of patients across different specimen types, thus lessening the issues of a single specimen as reference standard skewing detection towards 100%. These studies are primarily the 102/319 studies that were graded A in at least two specimens and are the only studies completely unrestricted in study inclusion. The 217 studies of B and C grade studies were included in the absence of A graded studies with multiple patients analyzed in more than one specimen. This was done to improve the accuracy of the detectability by avoiding the single specimen diagnostic reference standard as case confirmation was contingent upon positivity in that specimen. While this arbitrary sectioning limits the statistical capabilities, including the other excluded 2167 studies that were graded B and C in the presence of studies graded A would have been unfeasible. The novel categorizing method employed by this review preserves the under-studied specimen overview and is unlikely to be made another way with this scope.

However, CNS specimens present particular challenges for systematic assessment. Sampling is typically restricted to patients with severe or neurologically focused disease, often in the context of life-threatening illness or diagnostic uncertainty. This introduces a selection bias toward atypical or complicated cases, occasionally compounded by undiagnosed immunodeficiency or rare post-infectious syndromes, making generalization difficult. Moreover, viral RNA detection in CSF is frequently casuistic, reflecting low viral loads or transient CNS involvement. In less severe infections, lumbar puncture is rarely indicated, resulting in a paucity of comparative data. The infrequent collection and limited long-term storage of CSF further constrain its inclusion in standardized diagnostic or research protocols, hindering reproducibility and large-scale evaluation, as exemplified by the limited PCR detectability reported for neurotropic infections such as TBE virus and measles virus. Importantly, detection of viral RNA in CSF does not necessarily imply diagnostic utility. Rather, positivity in CNS specimens may provide insight into neuroinvasion, tissue tropism, or compartment-specific infection, as exemplified by HIV, where CSF RNA detection may reflect central nervous system involvement despite blood remaining the principal specimen for diagnosis and monitoring [352,353].

Post-study inclusion we incorporated the impending taxonomic changes to *Flaviviridae* based on Simmonds et al. 2025 for the included viruses and the suggested new family of *Hepaciviridae* [15]. The other suggested new family of *Pestiviridae* is, to our knowledge, as of yet not relevant in human disease and, as such, not relevant for the scope of the review.

Across the included viruses, significant variations were observed in detection rates among studies using similar specimen types for the same virus. These discrepancies may reflect virus-specific factors or methodological differences, such as variations in nucleic acid extraction methods, assay sensitivity or specificity, viral load (Ct or equivalent values), positivity thresholds, or the timing of specimen collection relative to disease progression. Another limitation is that NAAT detection in clinical specimens does not identify the cellular source of detected viral nucleic acids. Consequently, detection reflects the presence of viral genetic material within a specimen rather than the specific host cells or biological reservoirs contributing to that signal. Accordingly, this review evaluates specimen-level detectability rather than underlying cellular host interactions. A key limitation of this review is that such details were often poorly reported and, when available, the data were insufficient to allow meaningful comparison or adjustment for these factors. Furthermore, adherence to reporting frameworks such as MIQE was not consistently reported across studies and was therefore not amenable to systematic assessment.

## 5. Conclusions

This review underscores that the diagnostic application of NAATs across specimen types remains highly virus-specific and unevenly explored. For many pathogens, reliance on a single reference specimen—often blood or respiratory material—may overlook viable, less invasive alternatives. Broader validation of specimens such as urine or saliva could improve diagnostic accessibility in outbreak settings and support surveillance where conventional sampling is impractical. Alternative specimen types may also prove valuable in clinically complex situations, including when reference specimens are unavailable, collected outside the optimal diagnostic window, or yield negative results despite ongoing clinical suspicion. In such settings, knowledge of virus-specific detectability patterns across body sites may support diagnostic decision-making and improve case ascertainment. Importantly, knowledge of viral shedding kinetics across specimen types is essential for selecting the appropriate diagnostic test at different stages of disease, but this remains insufficiently defined for many human RNA viruses [354].

The marked heterogeneity between studies highlights the need for standardized reporting of key variables, including sampling timing, extraction methods, assay parameters, and Ct thresholds. Improved methodological transparency would enable cross-study comparability and support quantitative synthesis in future reviews.

Finally, the review identifies substantial evidence gaps for several virus–specimen combinations and limited longitudinal data on viral kinetics. Addressing these gaps through harmonized, cross-specimen studies could refine diagnostic algorithms, enhance outbreak preparedness, and inform future assay development across evolving viral taxonomies.

## Data Availability

The original contributions presented in this study are included in the article/Supplementary Material. Further inquiries can be directed to the corresponding author.

## Supplementary Materials

The following supporting information can be downloaded at: https://www.mdpi.com/article/10.3390/v18070743/s1, Table S1: PRISMA-ScR checklist; Table S2: Search strings and retrieval numbers; Table S3: Included study list and raw extracted data.

## Abbreviations

The following abbreviations are used in this manuscript:

NAAT	Nucleic acid amplification test
PBMC	Peripheral blood mononuclear cells

## Appendix A

**Table A1:** Evidence hierarchy categories.

Category	Description
A	Above 5 patients AND Multiple specimen types of interest from the same patients AND Consecutive sampling over time from patients untreated with targeted antivirals
B	Above 5 patients AND Multiple specimen types of interest from the same patients OR Consecutive sampling over time from patients untreated with targeted antivirals
C	No sample size requirement. At least one specimen type of interest with or without consecutive sampling.

For each virus and specimen combination, we checked full-text inclusions to identify category A studies. If available, only category A studies were included for that specimen type. If no category A studies were found, category B studies for that specific virus and specimen type were included. If neither category A nor B studies were identified, all full-text inclusions were included as category C studies.
